# Standardized nursing management of enzyme replacement therapy for late-onset Pompe disease

**DOI:** 10.1097/MD.0000000000024276

**Published:** 2021-01-22

**Authors:** Shan Tang, Jiachu Ma, Huaxing Meng, Junhong Guo, Shuyan Cao, Binquan Wang

**Affiliations:** a School of Nursing, Shanxi Medical University; b Department of Neurology, The First Hospital of Shanxi Medical University, Taiyuan, Shanxi, China.

**Keywords:** enzyme replacement therapy, Pompe disease, preparation method, standard operating procedure

## Abstract

Pompe disease or glycogen storage disease type II is a rare autosomal recessive disorder caused by a deficiency of the lysosomal enzyme a-glucosidase. Although enzyme replacement therapy (ERT) with 2 weekly intervals following was considered an effective treatment for Pompe disease in 2006, few patients can afford to receive treatment in China because of the high cost. This study aimed to examine the standard management of enzyme replacement therapy for late-onset Pompe disease among patients over the age of 14 years from a nursing perspective in order to assess operating procedures. ERT injection fluid dispensing and infusion procedures using different methods were analyzed and compared in 3 patients with advanced Pompe disease for forming standard operation procedures. In addition, the impact of different methods on time consumption was analyzed by 1-way analysis of variance. There were significant differences in time consumption between different dispensing and infusion methods. The time of dispensing and infusing the injection fluids using the cooperative method was 15.97 minutes shorter than that using the conventional method (95% CI: 4.51–27.43, *P *=* *.012); the time using the modified method was 20.93 minutes shorter than that using the conventional method (95% CI: 9.47–32.39, *P *=* *.012); and there was no significant difference between the cooperative and modified methods (*P *=* *.431). Enzyme replacement therapy entails frequent treatment and strict nursing requirements related to the intravenous infusion process. In this context, a standard operating procedure can be used to control nursing times and labor costs effectively while ensuring a safe and effective infusion process.

## 1 Introduction

Glycogen storage disease type II is a rare autosomal recessive progressive lysosomal storage disease due to mutations in the gene encoding α-glucosidase.^[1]^ As the disease was first reported by a Dutch pathologist Johannes Pompe in 1932, it is also called Pompe disease.^[2]^ According to the age of onset, affected organs, and the rate of disease progression, Pompe disease is classified into 2 major categories: infantile and late onset.^[3]^ In 2006, the acid α-glucosidase analog (Myozyme) was approved for clinical use as an enzyme replacement therapy (ERT).^[4]^ Since then, ERT has been used in many countries,^[1,5–10]^ including successful use in several pediatric cases in China. Although there have been many studies comparing the therapeutic outcomes of ERT in patients with Pompe disease, few studies have discussed details of nursing management during ERT. As a designated treatment facility for patients with Pompe disease over the age of 14 years in Shanxi Province, the First Hospital of Shanxi Medical University (Taiyuan, China) has summarized their experience and practice in standardizing the nursing management of recombinant human α-glucosidase replacement therapy in 3 patients. These standardized ERT nursing procedures ensure that the patients receive safe and effective routine treatment once every 2 weeks. The aim of the present study was to validate safety and effectiveness of the standardized ERT management procedures in late-onset patients with Pompe disease older than 14 years from the nursing perspective.

## 2 Materials & methods

### 2.1 Clinical case data

#### 2.1.1 General information

Included in this study were 3 patients with late-onset Pompe disease who received ERT at our department between April and July 2019. The diagnosis of the disease was confirmed on the basis of acid alpha-glucosidase activity through muscle biopsy and a skin fibroblast test.

The first patient was a 15-year-old girl weighing 32.5 kg, who was hospitalized mainly due to progressive aggravation of lower-limb weakness over a 7-year period accompanied by elevated levels of liver enzymes for 9 months. A neurological examination indicated that the patient had grade 2 muscle strength in her cervical flexor and extensor muscles, grade 4 strength in her double upper-limb proximal deltoid and biceps muscles, grade 4+ and 5 strength in her distal muscles, and grade 2 strength in her lower-limb proximal iliopsoas muscles. In addition, all 4 limbs exhibited positive tendon reflection. The cervical flexor, cervical extensor, and proximal limb muscles were all weak.

The second patient was a 22-year-old young woman weighing 39.5 kg with elevated levels of liver enzymes for approximately 20 years after catching a cold. The limb weakness became progressively worse over a 9-year period. A neurological examination indicated grade 2 strength in her cervical flexor and extensor muscles, grade 4 strength in both upper-limb deltoid and biceps muscles, grade 5 hand-grip, opposite finger, finger alignment, thumb-palm muscle, and little-finger palm muscle strength, grade 5 distal muscle strength, grade 2+ proximal lower extremity iliopsoas muscle strength, and grade 4 distal instep muscle flexion, flexor muscle, toe-back muscle flexion, and flexor muscle strength. In addition, the patient exhibited positive double upper-limb tendon reflection. There was also obvious atrophy in the patient's lower-limb and axonal muscles.

The third patient was a 20-year-old young man weighing 45 kg, who had to be hospitalized for 3 years due to 8-year weakness in both lower limbs and shortness of breath. A neurological examination indicated grade 3 cervical flexor and neck extensor muscle strength, grade 5 double upper-limb distal and proximal muscle strength, grade 2 lower-limb proximal muscle strength, and grade 5 lower-limb distal muscle strength. Further, the patient had double upper-limb and double-ankle reflexes (++) as well as positive knee reflexes. Moderate strength in the upper limbs, poor strength in the lower proximal limbs, and obvious axonal muscle atrophy were noted.

#### 2.1.2 Pre-treatment auxiliary examination

According to the guidelines for diagnosis, treatment, and management of Pompe disease,^[3,11]^ all 3 patients underwent arterial blood gas analysis, pulmonary function testing, cardiac color ultrasound, double lower-limb MRI, and electromyography to evaluate the cardiopulmonary function and muscle involvement before initializing the ERT procedures. The results showed abnormal liver function and serum creatine kinase (CK) level elevation. In addition, electromyography showed myogenic damage, and arterial blood gas analysis revealed carbon dioxide retention. All 3 patients had an extremely restrictive pulmonary ventilatory disorder but had normal lung volumes, residual gas volumes, and an elevated residual volume/total lung capacity ratio. The diffuse function was normal in both case 1 and 3, and moderate in case 2.

#### 2.1.3 Nursing evaluation

All 3 patients used the non-invasive ventilator-assisted breathing apparatus intermittently, with an activities-of-daily living (ADL) score of 80 to 95, which is known as an indicator of basic self-care competence. In addition, straight and elastic peripheral veins were noticed in the forearms and dorsum of hands in all patients, and no patient had a history of allergy.

### 2.2 Ethical considerations

This study was approved by the Ethics Committee of the First Hospital of Shanxi Medical University (Approval No. 2019-K037). Written informed consent was obtained from all 3 patients.

### 2.3 Treatment process

#### 2.3.1 Preparation materials

In accordance with the relevant drug instructions, preparations and materials included a disposable light-proof infusion set with a 24-G intravenous indwelling needle enclosed, a 0.2-micron low-protein combined in-line filter (these 3 families voluntarily purchased a Shimei extended infusion tube/filter manufactured by Taiwan United Medical Equipment Co., Ltd, as this tube/filter was not commercially available in Mainland China), other infusion materials (tourniquet, disinfectant cotton stick, skin disinfectant, infusion sticker), dexamethasone 5 mg, 100 mL 0.9% sodium chloride injection, a simple breathing balloon, a vacuum suction device, and other rescue materials.

#### 2.3.2 Procedure for calculation and preparation of the medicine

The number of bottles to dissolve was calculated by weight of each patient for a recommended dose of 20 mg/kg.^[8]^ If the calculated number was fractional, the measurement was rounded to the closest whole number. The required bottles were stored in a refrigerator set between 2°C and 8°C and returned to room temperature approximately 30 minute before dissolving.

Once the bottles were warmed, 50 mg alglucerase α was dissolved into each bottle along with 10.3 mL of water. The water was slowly added along the bottle wall to avoid making a strong impact with the powder to prevent foam formation. According to the instructions, each bottle was tilted and gently rolled without flipping, rotating, or vigorously shaking the bottle to avoid bubble formation, knowing that bubbles may affect the biological activity of Myozyme.

Immediately after dissolution, the preparation was diluted with 0.9% sodium chloride. Here, the final concentration of Myozyme was recommended to be 0.5 to 4.0 mg/mL. The solution was then slowly drawn from each bottle to avoid foam formation inside the syringe. Next, the solution was slowly injected into the sodium chloride solution, avoiding directly injecting the remaining air into the infusion bag to prevent foam formation. The infusion bag was then gently inverted or pressed to mix the diluents; shaking the bag was avoided.

#### 2.3.3 Procedures for medicine infusion

A regular intramuscular injection of dexamethasone 5 mg was given by using a pump to control the infusion rate. The procedure was initiated gently and accelerated slowly. It is recommended that the initial infusion rate not exceed 1 mg/kg/h. If the patient was unable to tolerate this, the rate was increased by 2 mg/kg/h every 30 minute until a maximum rate of 7 mg/kg/h was reached. At this rate, the infusion procedure took approximately 4 hours before it was completed in our patients (Table 1). The entire process was monitored by specialized personnel, who were also responsible for performing electrocardiography, measuring blood pressure, monitoring oxygen saturation, determining body temperature prior to infusion, and closely watching the patient for signs of allergic or hypersensitive reactions such as chills, headache, itchy skin, rash, rubella, and breathing difficulty. The procedure must be suspended if the patient develops a fever.^[12]^

**Table 1:** Enzyme replacement therapy for three patients with Pompe disease.

				Rate (mL/h)				
Patient	Weight (kg)	Volume (bottle)^∗^	Injection^†^ (mL)	Step 1	Step 2	Step 3	Step 4	Hour (h)
1	32.5	13	200	10	30	50	70	≈3.71
2	39.5	16	250	13	38	63	88	≈3.69
3	45	18	250	13	38	63	88	≈3.69

### 2.4 Experimental administration of medicine

Differences in dosing time between 3 different methods were compared using 1-way ANOVA (Table 2). All calculation times began once the 20 mL syringe package was opened and ended when the dilution was marked as complete (i.e., the medicine solution had been added to a 0.9% sodium chloride injection).

**Table 2:** Description and analysis of the ANOVA results.

Group	N	Mean ± Std (min)		Sum of squares	df	Mean square	F	Sig.
1	3	39.57 ± 5.03	Between groups	717.807	2	358.903	17.151	0.003
2	3	23.60 ± 5.77	Within groups	125.553	6	20.926		
3	3	18.63 ± 2.06	Total	843.360	8			

The conventional method (method 1) was conducted by a single nurse, who first used a 20 mL disposable sterile syringe (Shandong Weigao Group, specification 1.2 × 38 TWLB) to draw 10 mL sterile injection water to dissolve 1 bottle of the medicine, and then used a 1 mL disposable sterile syringe (Shandong Weigao Group, specification 0.45 × 16 RWLB) to add 0.3 mL sterile injection water to the solution bottle. This was repeated until the medicine was completely dissolved in all bottles.

The cooperative method (method 2) was completed by two nurses; one nurse used a 20 mL syringe to extract 10 mL of sterile injection water to dissolve 1 bottle of the medicine, and the other nurse used a 1 mL syringe to extract 0.3 mL sterile water for subsequent injection into the bottle. Like method 1, this was repeated for each bottle of medicine in sequence, with 1 nurse using a 20 mL syringe and the other using a 1 mL syringe until the medicine was completely dissolved in all bottles.

The modified method (method 3) was also completed by 2 nurses; each simultaneously used a 10 mL syringe to extract 10 mL of sterile injection water for use in dissolving all bottles of medicine for 1 patient. After all bottles had been injected with 10 mL of water, each nurse then used a 1 mL syringe to extract 0.3 mL of sterile water, which was separately added to each bottle. The solution was thus diluted in stages.

### 2.5 Statistical analysis

A 1-way ANOVA was conducted to compare differences in time used between the 3-drug dispensing and infusion methods. Each preparation method was used in all 3 patients.

A boxplot revealed no abnormal data. In addition, a Shapiro–Wilk test revealed that data from each patient were normally distributed (*P* > .05). Finally, a Levene homogeneity test of variance revealed that the data from each patient were squared (*P* *=* .246). All data are expressed as the mean ± standard deviation (SD).

## 3 Results

There were statistically significant differences in time between the 3 drug dispensing methods: [F(2,6) = 17.151, *P* = .003, w2 = 0.782]. The preparation time sequentially decreased for method 1 (M = 39.57, SD = 5.03), 2 (M = 23.60, SD = 5.77), and 3 (M = 18.63, SD = 2.06). The Tukey test results showed that the mean dosing time of method 1 was 15.97 minutes faster than that of method 2 (95% CI [4.51–27.43]; *P* = .012) and 20.93 minutes faster than that of method 3 (95% CI [9.47–32.39]; *P* = .003), and the mean dosing time of Method 2 was 4.97 minutes faster than that of method 3 (95% CI [–6.49–16.43]; *P* = .431) (Table 3).

**Table 3:** Tukey test results.

				95% Confidence interval	
Method (I)^†^	Method (J)^†^	Mean difference (I–J)^‡^	*P*	Lower bound	Upper bound
1	2	15.967^∗^	.012	4.5066	27.4267
	3	20.933^∗^	.003	9.4733	32.3934
2	3	4.967	.431	-6.4934	16.4267

## 4 Discussion

A standard operating procedure for ERT can facilitate overall safety and efficiency of the treatment. Our results indicated that the modified method reduced the mean waiting time from 30 minutes to 5 minutes when three patients were simultaneously treated. Related training programs should also be conducted to ensure proper results.

### 4.1 Perfect preparation is the basis for ensuring smooth treatment

Although the 3 patients receiving ERT in this study received single administrations and the total infusion volume did not exceed 250 mL, we found that the disposable steel intravenous infusion needles were suitable for use. Given the long infusion duration lasts approximately 4 hours, a peripheral venous indwelling needle is recommended to ensure safety, and a thinner and shorter catheter is especially preferred,^[13,14]^ knowing that this could minimize the risk of medicine exudation due to patient activity during the infusion process. It is not recommended to use a disposable infusion set if a polyvinyl chloride set can be used. At present, the European Union, the United States, Taiwan China, and Mainland China have all banned the use of Di-2-ethyhexyl Phthalate plasticized polyvinyl chloride infusion devices.^[15]^ Since different drug infusion devices may be made from different materials,^[16–18]^ it is recommended to use a thermoplastic elastomer infusion set, which contains no plasticizers, does not allow the adsorption of medicines, and guarantees medicinal efficacy. Use of thermoplastic elastomer infusion sets should be included in each hospital's standard operating procedures to avoid insufficient medicine concentrations resulting from the adsorption of infusion medicines by disposable infusion sets, thereby ensuring medicinal safety and effectiveness.

The infusion requires that the filtration infusion device be connected to a 0.2-micron low-protein combined in-line filter. Here, the tubing and filter media are different from those used in ordinary infusion sets. It is also difficult to successfully exhaust air from the infusion bag. However, the Murphy dropper in the infusion set can be turned upside down to form a U-shape. This slows the liquid flowing into the infusion tube and entering the filter. As such, the liquid can slowly and evenly make contact with the infusion tube, thereby avoiding the generation of small bubbles onto the attached wall, and allowing the liquid to completely wet the filter membrane, thus avoiding air retention. In addition, liquid waste is generated through both infusion and exhaust. To optimize the exhaust method, it is thus recommended to use a 0.9% sodium chloride injection to achieve ventilation before connecting the liquid source for the infusion process. The 0.9% sodium chloride injection should then be used to flush the tube after the infusion is completed, thus ensuring sufficient infusion of liquid and avoiding waste.

### 4.2 Tips and methods for formulating medicines: key points of difficulty during enzyme replacement therapy

The α-glucosidase drug injection used during ERT must be properly prepared to avoid foaming. This will reduce the risk of diminishing the activity of the medicine. To extract 10 mL of liquid medicine accurately during the procedure, it is necessary to use a 20 mL syringe to extract 10 mL of water, while a 1 mL syringe should be used to extract 0.3 mL of water, totaling 10.3 mL. When used for dissolving, the air inside the syringe should be evacuated; the tip of the needle should remain close to the bottle wall after inserting it into the bottle cap, thus creating a 45° angle. The water is then slowly injected. No vertical inversions are permitted at this time. This prevents the powder from falling into the solution and creating foam. Once the water is injected, the medicine is allowed to stand until it is completely dissolved into a clear liquid; any powder attached to the wall can be dissolved by tilting and gently tumbling the bottle. The dissolved solution should then be slowly extracted and directly injected into the saline solution bag for dilution; to avoid creating foam, this should not be injected into the air remaining in the infusion bag. The entire preparation process takes a considerable amount of time. In addition, repeated extraction of the liquid with the same syringe will increase the amount of friction between the syringe piston and the wall. Particles are also easily formed when puncturing the rubber plug of the bottle. Thus, the syringe should not be reused, and the user should avoid repeatedly puncturing the rubber plug through the same hole.

### 4.3 Standard operating procedures can effectively control the quality of cycle treatments

**Figure 1:**
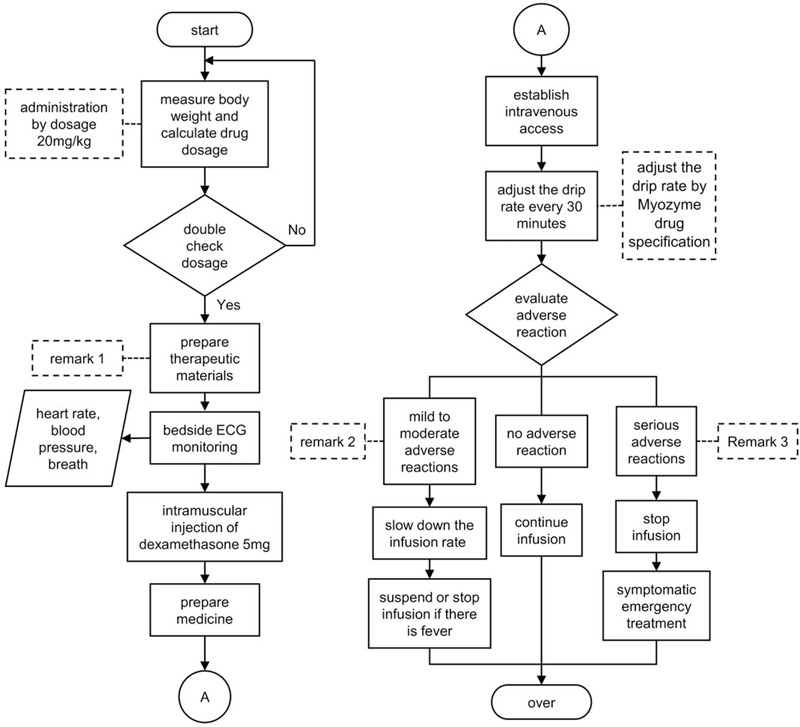
Standard operation procedures for Pompe disease enzyme replacement therapy. Remark 1. 1. Common materials: a disposable lightproof infusion set, a 24-G closed intravenous indwelling needle, a common treatment tray, and other infusion materials. 2. Special materials: a 0.2-micron low molecular weight protein combined with inline filter, dexamethasone, a simple breathing balloon, and a vacuum suction device. 3. Keep the drugs at room temperature. Remark 2. rash, fever, urticaria, facial flushing, hypertension, polypnea. Remark 3. 1. anaphylactic shock, cardiac arrest, dyspnea, polycardia, rapid onset asthma, or hypotension. 2. acute cardiopulmonary failure.

The standard operating procedures describe the work requirements in a uniform way according to the purpose of the operation, steps that must be taken, and operation requirements. These requirements serve as the guide and standard templates for daily work.^[19]^ This study demonstrated that application of ERT every 2 weeks prolonged the patient survival effectively and improved both motor and respiratory functions markedly in patients with late-onset Pompe disease (Fig. 1).^[20]^ In this context, Zhejiang Province, Shanxi Province, Qingdao City, and Tianjin City of China require that major illness insurance cover Pompe disease treatment using α-glucosidase, which solves the problem of high medical expenses on the part of the patient and makes it possible to popularize standardized ERT in the clinical setting on the part of the hospital. However, only 2 nurses in our hospital have received basic training for ERT. This is also the main reason why the treatment took a relatively long time in our patients. Our treatment was conducted by especially experienced members of the nursing staff. The overhead for intravenous infusion was calculated using the Resource-Based Relative Value Scale (RBRVS),^[21,22]^ in which labor costs (i.e., manpower cost = hours spent each time × relative value unit × conversion factor) accounted for a large portion. Therefore, standardization of the treatment process will shorten the amount of time needed to complete the treatment, thereby reducing the costs. Furthermore, an established work procedure can be used to train additional nursing staff to complete ERT independently. This will guarantee standard implementation practices and ensure quality control during the treatment process.

### 4.4 Limitations

This was a single-center study with a relatively small sample size and, therefore, may be subject to selection bias. Further investigations involving more patients from multiple centers should be conducted to verify the results. As patients were discharged immediately after treatment, only the adverse reactions during treatment were monitored. Despite the phone follow-up, whether a patient developed a delayed drug allergy after discharge or showed any sign of improvement after taking the medication was not tracked well, which could be improved.

## 5 Conclusions

ERT for Pompe disease is now covered by medical insurance in many provinces and cities in China. As such, it is becoming a routinized intravenous infusion care process. This study tested 3 dosage preparation methods to determine effectiveness and efficiency. Our results indicated that our methods 2 and 3, each utilizing 2 nurses, were most suitable for medication dispensing in clinical ERT. This finding is of clinical significance in controlling nursing labor costs. However, attention should also be paid to the quality of nursing care. Therefore, the standard operating procedures such as ours should be used in conducting ERT, knowing that they can strengthen current training procedures and ensure homogenized nursing-care quality as well.

## Acknowledgments

We would like to thank the 3 patients, from whom written informed consent has been obtained and for their active participation in this study.

## Author contributions

**Conceptualization:** Shan Tang, Binquan Wang.

**Data curation:** Jiachu Ma, Huaxing Meng.

**Formal analysis:** Junhong Guo, Shuyan Cao.

**Investigation:** Shan Tang.

**Methodology:** Shan Tang, Binquan Wang.

**Project administration:** Shan Tang.

**Supervision:** Binquan Wang.

**Validation:** Shan Tang.

**Writing – original draft:** Shan Tang.

**Writing – review & editing:** Shan Tang.
